# Urine D-ribose levels correlate with cognitive function in community-dwelling older adults

**DOI:** 10.1186/s12877-022-03288-w

**Published:** 2022-08-22

**Authors:** Xinyi Zhu, Yan Wei, Yingge He, Rongqiao He, Juan Li

**Affiliations:** 1 Center On Aging Psychology, Institute of Psychology, CAS Key Laboratory of Mental Health, Chinese Academy of Sciences, 16 Lincui Road, Chaoyang District, 100101 Beijing, China; 2 Department of Psychology, University of Chinese Academy of Sciences, Beijing, China; 3 State Key Laboratory of Brain and Cognitive Science, Institute of Biophysics, University of Chinese Academy of Sciences, 15 Datun Road, Chaoyang District, 100101 Beijing, China

**Keywords:** D-ribose, MMSE, Verbal fluency, Cognitive impairment, Older adults

## Abstract

**Background:**

D-ribose is involved in the pathogenesis of Alzheimer’s Disease. The study aimed to determine the association between D-ribose and cognitive function in a sample of community-dwelling older adults.

**Methods:**

A cross-sectional study was conducted in Chaoyang District, Beijing in 2019–2020. Eligible participants were community-based older adults aged 60 years and above. D-ribose was analyzed from the morning urine. Cognitive function, subjective cognitive decline, and depressive symptoms were measured by a battery of neuropsychological tests. Linear regressions were performed to determine the relationship between the urine D-ribose levels and cognitive performance.

**Results:**

A sample of 1725 participants (67.1% female) aged 60 to 85 years (69.40 ± 5.87 years, mean ± SD) was enrolled in the analysis. The urine D-ribose concentrations ranged from 1.53 to 208.89 μmol/L (median 38.10 μmol/L; interquartile range 22.52—64.96 μmol/L). Higher levels of D-ribose were associated with worse performance on Mini-Mental State Examination and verbal fluency when age, gender, education, depressive symptoms, and cardiovascular risk factors were included as covariates.

**Conclusions:**

The urine D-ribose was negatively correlated with cognitive function in community-dwelling older adults. The findings suggest that the dysmetabolism of D-ribose may play a role at the early stage of cognitive impairment.

**Supplementary Information:**

The online version contains supplementary material available at 10.1186/s12877-022-03288-w.

## Introduction

Besides D-glucose, D-ribose, as an essential component for energy production, is present in all living cells and plays important role in numerous biochemical processes [1, 2]. D-Ribose is extremely reactive in glycation with proteins such as neuronal tau [3] and α-synuclein [4], processing advanced glycation end products (AGEs), generating free oxygen radicals, and inducing protein aggregation [5]. It has been found that D-ribose is one of the major contributors to the glycation of serum protein and hemoglobin [6, 7], suggesting the involvement of D-ribose in diabetes.

Diabetes is a risk factor for dementia and mild cognitive impairment (MCI) [8]. A meta-analysis pooled data of 2.3 million individuals and found that diabetes was associated with a 60% increased risk of any dementia [9]. Cognitive impairment is common in diabetes patients, especially in Asia [10]. A recent meta-analysis estimated the pooled prevalence of MCI was 45% in patients with type 2 diabetes mellitus [10]. Since diabetes is closely associated with dementia [11], the relationship between D-ribose and dementia/cognitive impairment has been investigated. Intraperitoneal injection of D-ribose leads to cognitive impairment in mice with AGE formation in the brain [12]. Long-term gavage of D-ribose also can cause cognitive impairment in mice with amyloid-β deposition and Tau hyperphosphorylation in the brain [13]. In a case–control study [14], patients with Alzheimer’s Disease (AD) showed elevated urine D-ribose concentrations compared with age-matched controls, and the D-ribose concentration was negatively associated with global cognitive performance. Another study [15] reported a negative correlation between serum D-ribose and global cognition in type 2 diabetes mellitus patients with MCI, and the relationship remained after controlling for demographic, anthropometric, and blood clinical covariates. D-ribose is thought to be involved in the pathogenesis of AD [16].

Although the impacts of D-ribose on cognitive impairment have been reported in laboratory studies and case–control studies with patients with AD and MCI, it is unclear whether the association between D-ribose and cognition exists in community-based samples. Thus, the current study aims to determine the relationship between urine D-ribose and cognitive functioning in a large sample of community-dwelling older adults.

## Methods

### Design and setting

A cross-sectional study was conducted in Chaoyang District, Beijing in 2019–2020 in collaboration with the Chaoyang District Center for Disease Control and Prevention. A multistage sampling method was used, with community medical centers as the cluster units. There are 41 community medical centers in the Chaoyang District. Medical centers which have more than 7000 registered older residents and have available space for the study were considered eligible. Ten eligible medical centers were enrolled in the study and a sample of about 200 older adults was randomly selected in each medical center.

The study was a part of “A Community-Based Stepped Screening and Intervention Project on Age-related Cognitive Impairment”, which was prospectively registered at the Chinese Clinical Trial Registry on August 28, 2019 (ChiCTR, www.chictr.org.cn; ChiCTR1900025487). The study protocol was approved by the Ethics Committee of the Institute of Psychology, Chinese Academy of Sciences (H19019). All participants provided written informed consent.

### Participants

A sample of 2136 participants was finally recruited. Eligible participants met the following inclusion criteria: (1) ≥ 60 years old; (2) community-dwelling. The exclusion criteria were: (1) severe neurological, psychiatric, or medical conditions based on self-reports, such as Parkinson’s Disease, Alzheimer’s Disease, or other dementia; (2) severe loss of vision, hearing, or communicative abilities which would hinder the participation of the study. Each participant underwent a structured face-to-face interview at community medical centers. The interviews were administered by community doctors, nurses, or college student volunteers who had been specially trained for the study.

**Fig. 1:**
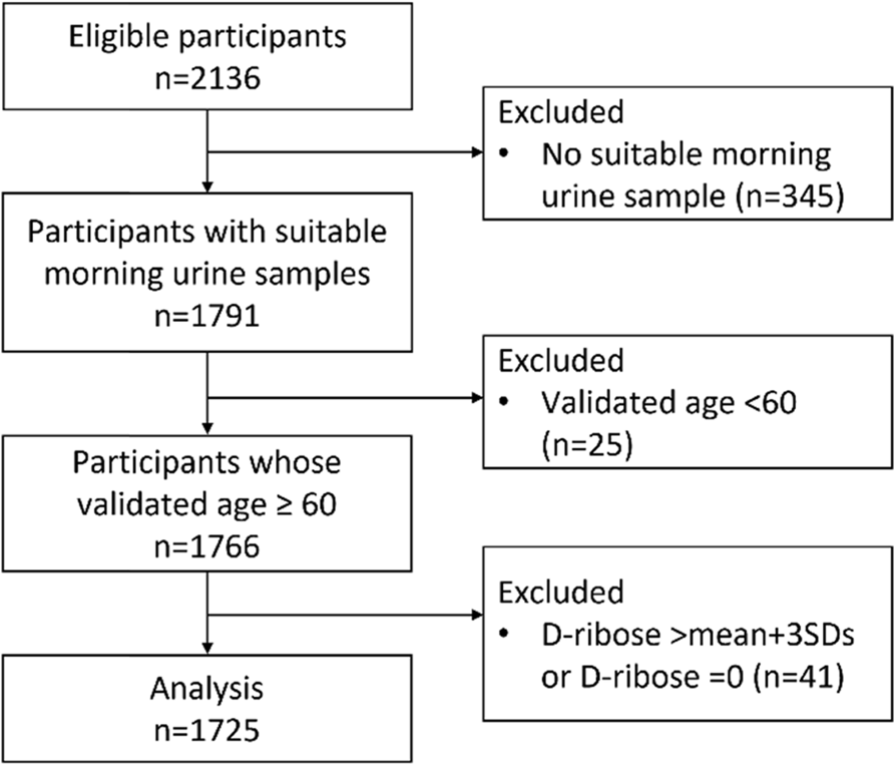
Flow chart of participants

Within four weeks after the interview, urine samples were collected. Participants were required to take aliquots of the first urine in the morning before water intake and breakfast. Participants were asked to avoid consumption of fatty and spicy foods and rigorous exercise for one week before sampling. All urine samples were stored in a sealed sterile container at -80℃ until analysis. Out of 2136 participants, a sample of 1791 participants successfully provided suitable morning urine samples. Next, participants whose validated age was younger than 60 were excluded from the analysis (*n* = 25). Urine D-ribose concentrations ≥ mean + 3SDs and D-ribose concentrations = 0 were then treated as outliers and excluded from analysis (*n* = 41). Finally, data of 1725 participants were included in the analysis. Figure 1 shows the flow of participants.

### Measurements

A battery of neuropsychological tests was administered to all participants. Cognitive function was assessed in five domains: (1) global cognition (Mini-Mental State Examination, MMSE [17]); (2) episodic memory (Paired Associative Learning Test from the Clinical Memory Scale [18]); (3) working memory (Digit Span Backward Task from the Wechsler Adult Intelligence Scale-Chinese Revision [19], WAIS-RC); (4) executive function (Category Fluency Task [20]); (5) processing speed (Digit Symbol Substitution Test from the WAIS-RC [19], DSST). The AD8 questionnaire was used to measure subjective cognitive decline [21], with a higher score suggesting more subjective cognitive decline.

Covariates included demographic characteristics (age, gender, and education years), depressive symptoms, and cardiovascular risk factors, which are recognized risk factors for late-life cognitive decline [22]. Depressive symptoms were measured using the Center for Epidemiological Studies Depression (CES-D) Scale [23], with a higher score indicating more depressive symptoms. Cardiovascular risk factors included self-reported hypertension, heart disease, diabetes, hyperlipidemia, stroke, current smoking, current drinking, and physical inactivity. Participants reported their frequency (never, monthly, weekly, 3–4 days/week, 5–6 days/week, daily) of participation in four moderate-intensity physical activities (dance, Taichi, cycling, and other self-report activities). The frequency of participation in each activity was coded as 0 for none and monthly, 1 point for weekly, 3.5 for 3–4 days/week, 5.5 for 5–6 days/week, and 7 for daily. Participants who scored 0 on physical activity were categorized as physical inactivity.

### Analysis of urine D-ribose

Analysis of urine D-ribose was performed in a double-blind manner. Urine D-ribose was measured as previously described [7, 24]. Urine samples (thawed at 4 °C) were centrifuged (12,000 rpm, 4 °C, 10 min). A 0.4 mL aliquot of the supernatant was mixed with 0.6 mL 4-(3-methyl-5-oxo-2-pyrazolin-1-yl) benzoic acid (MOPBA, final concentration 150 mM in 250 mM NaOH in 50% methanol–water solution, Sigma Aldrich, USA) and then heated in a 70 °C water bath for 90 min, followed by additional centrifugation (12,000 rpm, 4 °C, 10 min). The mixture was acidified by 150 μL 2 M HCl solution, centrifuged (12,000 rpm, 4 °C, 10 min), and filtered through 0.22 μm membranes. Twenty microliters of the solution were subjected to high-performance liquid chromatography (HPLC, LC-20A, Shimadzu, Japan) system with an ultraviolet detector. The MOPBA-sugar derivative was collected from the C18 column with a binary mobile phase gradient. Mobile phase A was 10 mM sodium 1-hexanesulfonate (pH 2.5, Tokyo Chemical Industry, Japan), and mobile phase B was a 50% acetonitrile solution. The elution conditions were 38%–60% B for 15 min, 100% B for 5 min, and 38% B for 5 min. The flow rate was 1 ml/min, and the column temperature was 40 °C. The reference concentrations of D-ribose were determined according to the standard curves.

### Statistical analysis

The Shapiro–Wilk Test for normality showed that the concentrations of urine D-ribose were not normally distributed (statistic = 0.84, df = 1725, *p* < 0.001). As the concentrations of urine D-ribose were positively skewed (skewness = 2.74, SE of skewness = 0.06), bivariate correlations among D-ribose, demographic variables, and cognitive measures were calculated by Spearman’s rank correlation. The urine D-ribose concentrations were divided into four levels based on the quartiles: first quarter (< 22.52 μmol/L, n = 431), second quarter (22.52–38.10 μmol/L, n = 431), third quarter (38.10–64.96 μmol/L, *n* = 432), and fourth quarter (> 64.96 μmol/L, *n* = 431). One-way analysis of variance (ANOVA), the Kruskal–Wallis test, or Pearson’s Chi-squared test was used to compare group differences related to the D-ribose levels in demographic variables, cardiovascular risk factors, and cognitive performance when appropriate. As multiple correlations and multiple comparisons were conducted across five cognitive tests, Bonferroni correction was adopted to control the family-wise error rate. Thus, the significance level was set at 0.05/5 = 0.01 in correlation tests and ANOVAs for cognitive tests.

Multiple linear regressions were further conducted to determine the strength of the relationships between the urine D-ribose levels and cognitive scores. Results from three models were reported: Model 1 included only the D-ribose levels; Model 2 was adjusted for demographic characteristics (age, gender, and education); in Model 3, additional adjustment was made for depressive symptoms and cardiovascular risk factors. The covariates included are recognized risk factors for late-life cognitive decline [22].

To validate the relationship between the urine D-ribose and cognitive functions, three secondary analyses were further performed. First, analyses were repeated for all urine D-ribose data with outliers (*n* = 1766). Second, the D-ribose concentration was treated as a continuous variable in separate linear regressions. Third, cognitive scores were treated as dichotomous dependent variables and logistic regressions were performed. MMSE scores were recoded into a dichotomous variable (cognitive impairment) according to education-based cutoffs widely accepted and used in China [25] (< 18 for participants with no formal education, < 21 for participants with 1–6 years of education, and < 25 for participants with more than 6 years of education). Verbal fluency scores were coded as dichotomous using a cutoff score of < 16 [26]. Covariates were the same as variables included in the linear regression. All statistical analyses were performed using IBM SPSS Statistics 21 for Windows (IBM Corp., NY).

## Results

### Sample characteristics and urine D-ribose

Table 1 summarizes information on demographics, cognitive performance, depressive symptoms, and cardiovascular risk factors of the sample. The sample included 1725 participants (67.1% female) aged 60 to 85 years (69.40 ± 5.87, mean ± SD). The urine D-ribose concentrations ranged from 1.53 to 208.89 μmol/L (38.10, 22.52—64.96; median, interquartile range). The urine D-ribose concentrations were negatively correlated with age (Spearman *r* = -0.12, *p* < 0.001; Table 2) but not education years. The female participants showed a lower level of urine D-ribose than the male (female 35.01, 22.52—64.96; male 44.63, 26.76—78.42; *p* < 0.001).

**Table 1:** Demographics and cognitive scores in different urine D-ribose levels

	**Whole sample**	**1st quarter**	**2nd quarter**	**3rd quarter**	**4th quarter**	***P*-value**
	(*n* = 1725)	(*n* = 431)	(*n* = 431)	(*n* = 432)	(*n* = 431)	
**Urine D-ribose (μmol/L)**^a^	38.10 (22.52–64.96)	15.09 (11.21–19.09)	29.14 (25.59–33.42)	48.87 (42.97–55.65)	93.76 (74.84–124.22)	< 0.001
**Age**	69.40 ± 5.87	70.36 ± 5.90	69.56 ± 5.76	69.09 ± 5.96	68.60 ± 5.73	< 0.001
**Education years**	10.11 ± 3.49	10.30 ± 3.41	10.16 ± 3.28	10.15 ± 3.67	9.84 ± 3.56	0.26
**Female**^b^	67.1%	75.6%	68.4%	69.2%	55.0%	< 0.001
**MMSE**^c^	27.31 ± 2.68	27.66 ± 2.50	27.22 ± 2.65	27.22 ± 2.81	27.12 ± 2.75	0.015
**PALT**^c^	7.37 ± 4.06	7.65 ± 4.23	7.25 ± 3.96	7.23 ± 4.08	7.34 ± 3.98	0.42
**Digit Span Backward**^c^	4.48 ± 1.49	4.58 ± 1.53	4.46 ± 1.48	4.52 ± 1.56	4.36 ± 1.37	0.19
**Verbal Fluency**^c^	19.33 ± 5.39	20.22 ± 5.48	19.38 ± 5.56	19.39 ± 5.40	18.33 ± 4.93	< 0.001
**Digit Symbol**^c^	29.73 ± 11.74	30.07 ± 11.72	29.60 ± 12.92	30.44 ± 11.41	28.81 ± 10.81	0.2
**AD8**	1.83 ± 1.75	1.85 ± 1.69	1.80 ± 1.69	1.87 ± 1.86	1.80 ± 1.74	0.91
**CES-D**	6.81 ± 8.23	7.58 ± 8.46	6.56 ± 7.61	7.19 ± 8.78	5.89 ± 7.97	0.001
**Current smoking**^b^	10.3%	5.6%	10.0%	8.8%	16.7%	< 0.001
**Current drinking**^b^	12.7%	7.2%	11.4%	12.0%	20.2%	< 0.001
**Physically inactive**^b^	12.7%	7.2%	11.4%	12.0%	20.2%	0.001
**Hypertension**^b^	56.2%	59.2%	55.2%	53.0%	57.3%	0.3
**Heart disease**^b^	31.7%	33.6%	34.3%	29.9%	29.0%	0.24
**Diabetes**^b^	28.9%	29.5%	27.6%	26.6%	32.0%	0.32
**Hyperlipidemia**^b^	40.6%	43.4%	38.1%	39.8%	41.3%	0.43
**Stroke**^**b**^	10.7%	10.4%	10.0%	10.9%	11.4%	0.92
**Medication for hypertension**^b^	52.8%	56.1%	52.0%	50.7%	52.4%	0.42
**Medication for diabetes**^b^	26.4%	25.8%	25.8%	23.8%	30.2%	0.19
**Medication for hyperlipidemia**^b^	30.0%	32.3%	28.5%	30.1%	29.2%	0.66

Data was shown as mean ± SD and analyzed using ANOVA except where noted

*MMSE* Mini-Mental State Examination, *PALT* Paired Associative Learning Test, *CES-D* Center for Epidemiological Studies Depression Scale

^a^Data was shown as median (interquartile range) and analyzed using the Kruskal–Wallis Test

^b^Data was shown as percentage and analyzed using the Mann–Whitney U Test

^c^The significance level was set at 0.01 to control family-wise error rate (Bonferroni correction)

**Table 2:** Correlations between urine D-ribose, demographics, and cognitive scores

	D-ribose	Age	Education	MMSE^a^	PALT^a^	Digit Span Backward^a^	Verbal Fluency^a^	Digit Symbol^a^	AD8	CES-D
D-ribose	1.00	-0.12^**^	-0.04	-0.08^**^	-0.02	-0.03	-0.10^***^	-0.03	-0.02	-0.10^***^
Age		1.00	-0.02	-0.12^***^	-0.18^***^	-0.14^***^	-0.14^***^	-0.32^***^	0.15^***^	0.13^***^
Education			1.00	0.33^***^	0.20^***^	0.31^***^	0.39^***^	0.42^***^	-0.15^***^	0.01
MMSE^a^				1.00	0.40^***^	0.41^***^	0.40^***^	0.38^***^	-0.16^***^	-0.09^***^
PALT^a^					1.00	0.36^***^	0.35^***^	0.33^***^	-0.06^**^	-0.17^***^
Digit Span Backward^a^							0.37^***^	0.39^***^	-0.10^***^	-0.06^**^
Verbal Fluency^a^							1.00	0.52^***^	-0.13^***^	-0.08^***^
Digit Symbol^a^								1.00	-0.15^***^	-0.06
AD8									1.00	0.36^***^
CES-D										1.00

*MMSE* Mini-Mental State Examination, *PALT* Paired Associative Learning Test, *CES-D* Center for Epidemiological Studies Depression Scale

^a^The significance level was set at 0.01

^**^p < 0.01

^***^p < 0.001

### Urine D-ribose and cognitive performance

The bivariate correlations between the urine D-ribose concentrations and cognitive performance were shown in Table 2. The urine D-ribose concentrations were negatively correlated with MMSE (Spearman *r* = -0.08, *p* < 0.01) and verbal fluency (Spearman *r* = -0.10, *p* < 0.001). The urine D-ribose concentrations were also correlated with depressive symptoms (Spearman *r* = -0.10, *p* < 0.001).

**Fig. 2:**
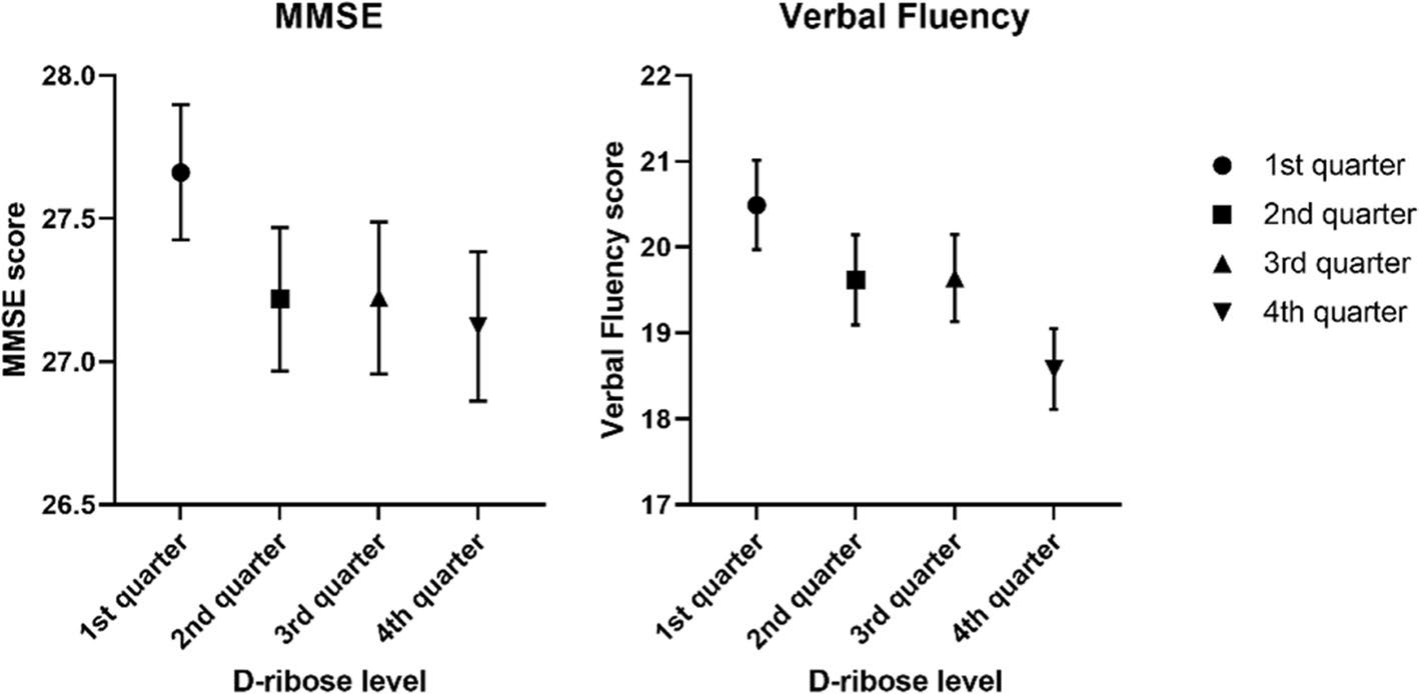
Means and 95% confidence intervals of MMSE and verbal fluency scores in four quartiles of D-ribose

ANOVA results demonstrate that group differences were observed in MMSE and verbal fluency across four D-ribose levels, but the difference in MMSE (*p* = 0.015) was not significant after the Bonferroni correction (Table 1). Post hoc multiple comparisons (Bonferroni procedure) showed that participants in the lowest D-ribose level had higher MMSE scores compared with their counterparts in the highest level of D-ribose (*p* = 0.02). Regarding verbal fluency, participants in the highest D-ribose level significantly scored lower than the other three quarters (4^th^ quarter vs. 1^st^ quarter, *p* < 0.001; 4^th^ quarter vs. 2^nd^ quarter, *p* = 0.02; 4^th^ quarter vs. 3^rd^ quarter, *p* = 0.02). Means and 95% confidence intervals (CI) of MMSE and verbal fluency scores in four quartiles are shown in Fig. 2.

### Urine D-ribose levels predict cognitive performance

As significant bivariate correlations and group differences across D-ribose levels have been observed in MMSE and verbal fluency, multivariate linear regressions were further conducted for the two tests. Table 3 summarizes the results of linear regressions. Higher levels of urine D-ribose significantly predicted lower MMSE and verbal fluency (Model 1), and the relationship remained significant when demographic variables, cardiovascular risk factors, and depressive symptoms were controlled (Models 2 and 3). Compared with participants in the lowest level of D-ribose, their counterparts in the other three levels scored about a half-point less in MMSE. For verbal fluency, compared with participants in the lowest level, older adults in the 2^nd^ quarter and 3^rd^ quarter of D-ribose scored 0.79 and 0.84 fewer points while those in the highest level scored 1.72 points less.

**Table 3:** Association between D-ribose levels and MMSE/verbal fluency using linear regression

	**Model 1**		**Model 2**		**Model 3**	
	**Beta**^**a**^	**SE**	**Beta**^**a**^	**SE**	**Beta**^**a**^	**SE**
**Dependent Variable: MMSE**						
D-ribose level (1st quarter)	(reference)		(reference)		(reference)	
D-ribose level (2nd quarter)	-0.45*	0.18	-0.45**	0.17	-0.46**	0.16
D-ribose level (3rd quarter)	-0.44*	0.18	-0.48**	0.17	-0.47**	0.16
D-ribose level (4th quarter)	-0.55**	0.18	-0.49**	0.17	-0.51**	0.17
Age			-0.08***	0.01	-0.08***	0.01
Male			-0.33**	0.13	-0.18	0.15
Education			0.30***	0.02	0.29***	0.02
CES-D					-0.03***	0.01
Physical inactivy					-0.08	0.12
Current smoking					-0.23	0.22
Current drinking					-0.12	0.20
Hypertension					-0.08	0.12
Heart disease					0.25	0.13
Diabetes					-0.07	0.13
Hyperlipidemia					0.52***	0.12
Stroke					-0.15	0.19
**Dependent Variable: Verbal Fluency**						
D-ribose level (1st quarter)	(reference)		(reference)		(reference)	
D-ribose level (2nd quarter)	-0.82*	0.37	-0.82*	0.33	-0.79*	0.33
D-ribose level (3rd quarter)	-0.83*	0.36	-0.87**	0.33	-0.84*	0.33
D-ribose level (4th quarter)	-1.88***	0.37	-1.71***	0.34	-1.72***	0.34
Age			-0.13***	0.02	-0.13***	0.02
Male			-0.64**	0.26	-0.31	0.30
Education			0.60***	0.03	0.59***	0.03
CES-D					-0.03***	0.01
Physical inactivy					0.22	0.24
Current smoking					-1.26**	0.43
Current drinking					-0.20	0.40
Hypertension					-0.44	0.25
Heart disease					-0.001	0.26
Diabetes					-0.08	0.27
Hyperlipidemia					0.90***	0.25
Stroke					-0.43	0.38

*CES-D* Center for Epidemiological Studies Depression Scale

^a^Unstandardized coefficients

^*^p < 0.05

^**^p < 0.01

^***^ *p* < 0.001

### Sensitivity analysis

When including all D-ribose data with outliers (*n* = 1766), results remained the same. The D-ribose levels were negatively associated with MMSE and verbal fluency (Tables S1, Additional file 1).

When the urine D-ribose was treated as a continuous variable in linear regression, D-ribose was negatively associated with verbal fluency (*β* = -0.01, *p* < 0.001) when age, gender, education years, depressive symptoms, and cardiovascular risk factors were included as covariates. The associations between D-ribose and MMSE became marginally significant (MMSE, *β* = -0.003, *p* = 0.059).

When using dichotomous dependent variables, logistic regressions showed that D-ribose levels were negatively associated with cognitive impairment and verbal fluency. In specific, compared to the 1^st^ quarter of D-ribose, the odds ratio of cognitive impairment was significantly higher for the 2^nd^ quarter (OR: 2.03; 95% CI: 1.24–3.30). The odds ratio of verbal fluency < 16 was higher for participants in the 2^nd^ quarter (OR: 1.88; 95% CI: 1.33–2.65) and 4^th^ quarter (OR: 1.89; 95% CI: 1.33–2.68) of D-ribose.

## Discussion

The current study revealed a negative association between the urine D-ribose levels and cognition in a large sample of community-dwelling older adults. To our knowledge, the current study provides the first community-based evidence on the relationship between D-ribose and cognitive functioning.

Older adults with higher levels of D-ribose performed worse on MMSE and verbal fluency, compared with those with the lowest level of D-ribose. The findings are consistent with the previous studies which observed elevated D-ribose in AD patients [14] and diabetes patients with MCI [15]. The current study extends the negative relationship between D-ribose and cognitive function from small clinical samples to a large community-based sample. Adjustment for covariates including demographic variables, depressive symptoms, and cardiovascular risk factors did not weaken the strength of the correlations. The results suggest the robustness of the negative association between D-ribose and cognitive function in community-dwelling older adults. As the test for the urine D-ribose is noninvasive, convenient, and fast, the results implicate the potential of D-ribose to serve as a biomarker for the progression of cognitive impairment.

Unlike previous studies of D-ribose using a single cognitive measure such as MMSE and the Montreal Cognitive Assessment, our study examined whether D-ribose was linked with different aspects of cognitive function. The relationship between D-ribose and cognition was only observed in MMSE and verbal fluency but not tests for episodic memory, working memory, or processing speed. MMSE is a brief global cognition test widely used to screen cognitive impairment and dementia [17]. Verbal fluency, a task that requires participants to rapidly generate exemplars from a specified category, is often considered as a measure of executive function [27]. Verbal fluency is sensitive to cognitive impairment and a predictor of progression to AD [28–30]. The correlations may suggest that the dysmetabolism of D-ribose may play a role at a very early stage of cognitive impairment. Besides global cognition, elevated D-ribose was associated with worse executive functioning in the current study. Impaired executive function has been widely reported in patients with diabetes mellitus [31], but diabetes patients also show deficits in other cognitive domains including episodic memory, attention, and processing speed [32]. It may suggest that executive function is sensitive to dysmetabolism of D-ribose, but more studies are needed to examine the reliability of the selective association between D-ribose and executive function.

We would like to consider that cognitive ability is related to D-ribose metabolism. This viewpoint is based on the following observations: (1) D-Ribose dysmetabolism, such as diabetes, can occur from early adulthood to agedness in one’s life [33]. (2) Brain metabolic dysfunction is believed at the core of AD [34]. Inherent changes in bioenergetics profiles were associated with late-onset AD [35]. Johnson and collaborators carried out a large-scale proteomic analysis of AD brain and cerebrospinal fluid and revealed early changes in energy metabolism associated with microglia and astrocyte activation [36]. Participating in energetic metabolism is the most important function of D-ribose, for instance as a component of the synthesis of ATP. (3) Ribosylation rapidly produces AGEs, which are regarded as one of the most important risk factors for cognitive impairment [37, 38]. (4) Endogenous D-ribose level is associated with AD at the clinical stage [14], and D-ribose is thought to be a pathogen for AD [5, 16]. Finally, (5) The reason that D-ribose is probably used as a biomarker to predict cognitive performance in community-dwelling older adults is that the pentose can be correctly determined in urine samples with the HPLC [24]. It has to be explained that we cannot measure the blood D-ribose with this method because D-ribose is rapidly reacted with serum proteins in blood samples, leading to deviations of the resultant data. However, the urine contains very few proteins, which consumes much less D-ribose than blood, and makes the data in a trusted range.

The strength of the study includes large sample size and multiple cognitive measures. Several limitations should also be mentioned. First, because of the cross-sectional design of the study, we cannot conclude the temporal relationship between D-ribose and cognition or make a causal inference. It would be meaningful to collect longitudinal data to examine whether D-ribose predicts future cognitive decline or incidence of cognitive impairment. Second, no biochemical index besides D-ribose was collected in the current study. Including more biological indicators may help to explain the mechanism underlying the inverse relationship between D-ribose and cognition.

## Conclusions

The urine D-ribose levels were negatively correlated with cognitive function in community-dwelling older adults. The findings suggest that the dysmetabolism of D-ribose may play a role at the early stage of cognitive impairment.

## Supplementary Information

**Additional file: TableS1.** Association betweenD-ribose levels and MMSE/verbal fluency in sample with D-ribose outliers (*n*=1766).

## Data Availability

The datasets used and analyzed during the current study are available from the corresponding author (Juan Li) on reasonable request.

## Abbreviations

AD: Alzheimer’s Disease
AGEs: Advanced glycation end products
ANOVA: Analysis of variance
CES-D: Center for Epidemiological Studies Depression Scale
HPLC: High-performance liquid chromatography
MCI: Mild cognitive impairment
MMSE: Mini-Mental State Examination
MOPBA: 4-(3-Methyl-5-oxo-2-pyrazolin-1-yl) benzoic acid
WAIS-RC: Wechsler Adult Intelligence Scale-Chinese Revision
