# Supplementary material for: Urine D-ribose levels correlate with cognitive function in community-dwelling older adults
Source: BMC Geriatr. 2022 Aug 22;22:693. doi: 10.1186/s12877-022-03288-w (PMC9396817; doi:10.1186/s12877-022-03288-w)
Supplement: Supplementary file 1 — Additional file: TableS1. Association betweenD-ribose levels and MMSE/verbal fluency in sample with D-ribose outliers (n=1766). [file 12877_2022_3288_MOESM1_ESM.docx]

**Table S1.** Association between D-ribose levels and MMSE/verbal fluency in sample with D-ribose outliers (n=1766)

|  | **Model 1** | |  | **Model 2** | |  | **Model 3** | |
| --- | --- | --- | --- | --- | --- | --- | --- | --- |
|  | **Beta** | **SE** |  | **Beta** | **SE** |  | **Beta** | **SE** |
| Dependent Variable: MMSE |  |  |  |  |  |  |  |  |
| D-ribose level (1st quarter) | (reference) |  |  | (reference) |  |  | (reference) |  |
| D-ribose level (2nd quarter) | -0.45* | 0.18 |  | -0.46** | 0.17 |  | -0.47** | 0.16 |
| D-ribose level (3rd quarter) | -0.45* | 0.18 |  | -0.49** | 0.17 |  | -0.48** | 0.16 |
| D-ribose level (4th quarter) | -0.56** | 0.18 |  | -0.49** | 0.17 |  | -0.49** | 0.17 |
| Age |  |  |  | -0.09*** | 0.01 |  | -0.08*** | 0.01 |
| Male |  |  |  | -0.33** | 0.13 |  | -0.18 | 0.15 |
| Education |  |  |  | 0.30*** | 0.02 |  | 0.30*** | 0.02 |
| CES-D |  |  |  |  |  |  | -0.03*** | 0.01 |
| Physical inactivy |  |  |  |  |  |  | -0.06 | 0.12 |
| Current smoking |  |  |  |  |  |  | -0.31 | 0.22 |
| Current drinking |  |  |  |  |  |  | -0.05 | 0.20 |
| Hypertension |  |  |  |  |  |  | -0.10 | 0.12 |
| Heart disease |  |  |  |  |  |  | 0.24 | 0.13 |
| Diabetes |  |  |  |  |  |  | -0.08 | 0.13 |
| Hyperlipidemia |  |  |  |  |  |  | 0.54*** | 0.12 |
| Stroke |  |  |  |  |  |  | -0.22 | 0.19 |
| Dependent Variable: Verbal Fluency |  |  |  |  |  |  |  |  |
| D-ribose level (1st quarter) | (reference) |  |  | (reference) |  |  | (reference) |  |
| D-ribose level (2nd quarter) | -0.84* | 0.37 |  | -0.83* | 0.33 |  | -0.81* | 0.33 |
| D-ribose level (3rd quarter) | -0.84* | 0.36 |  | -0.88** | 0.33 |  | -0.85* | 0.33 |
| D-ribose level (4th quarter) | -1.80*** | 0.36 |  | -1.60*** | 0.33 |  | -1.59*** | 0.33 |
| Age |  |  |  | -0.13*** | 0.02 |  | -0.13*** | 0.02 |
| Male |  |  |  | -0.64** | 0.25 |  | -0.32 | 0.30 |
| Education |  |  |  | 0.60*** | 0.03 |  | 0.58*** | 0.03 |
| CES-D |  |  |  |  |  |  | -0.05*** | 0.01 |
| Physical inactivy |  |  |  |  |  |  | 0.21 | 0.24 |
| Current smoking |  |  |  |  |  |  | -1.10** | 0.42 |
| Current drinking |  |  |  |  |  |  | -0.09 | 0.39 |
| Hypertension |  |  |  |  |  |  | -0.45 | 0.25 |
| Heart disease |  |  |  |  |  |  | -0.04 | 0.26 |
| Diabetes |  |  |  |  |  |  | 0.13 | 0.27 |
| Hyperlipidemia |  |  |  |  |  |  | 0.85*** | 0.25 |
| Stroke |  |  |  |  |  |  | -0.47 | 0.38 |

*Notes*: CES-D: Center for Epidemiological Studies Depression Scale.

^a^ Unstandardized coefficients.

^*^*p* <0.05, ^**^*p* <0.01, ^***^ *p* <0.001
